# Assessing young children's national identity through human-computer interaction: A game-based assessment task

**DOI:** 10.3389/fpsyg.2022.956570

**Published:** 2022-09-28

**Authors:** Xiumin Hong, Qianqian Liu

**Affiliations:** Institute of Early Childhood Education, Faculty of Education, Beijing Normal University, Beijing, China

**Keywords:** game-based assessment, human-computer interaction, national identity, young children, reliability

## Abstract

As a way of human-computer interaction, game-based assessment is more suitable for young children because it is situational, interesting, and effective. National identity is an important factor affecting the overall development of young children and the future development of a country, which has attracted extensive attention from researchers. Nevertheless, the assessment of young children's national identity is still based on traditional evaluation, including questionnaires and interviews, which have the limitations of being inaccurate, dull, and time-consuming. To understand the characteristics of children's national identity, it is necessary to use scientific and interactive assessment methods. The present study investigated whether the game-based assessment we developed specifically would be an appropriate tool to measure young children's national identity. The results show that the game-based assessment had good item discrimination. Exploratory factor analysis demonstrated the game covered three aspects: national cognition mastery, national emotion engagement, and national behavior tendency. The confirmatory factor analysis suggested that the model with three factors fit the data well. The internal consistency, the split-half reliability, and the test-retest reliability meet standards. Overall, the results indicated that this game can be successfully used to assess young children's national identity with acceptable validity and reliability. Our study provides strong evidence for the use of human-computer interaction in child measurement. These findings are the first to demonstrate the promise of game-based assessment in assessing children's national identity reliably and effectively.

## Introduction

Over the past few decades, the assessment of human-computer interaction has become one of the topics most studied in the field of psychology (Zhang and Na, 2004; Tapingkae et al., 2020). The game-based assessment, as a way of human-computer interaction, is more appropriate for children for assessment as it is situational, interesting, and effective. As an important factor, the national identity is influential in children's all-around development and the country's future, so it has attracted extensive attention from researchers (Brown, 2011; Murphy and Janet, 2013; Violante et al., 2019). However, children's national identity is still assessed based on traditional methods such as questionnaires and interviews, and this kind of assessment is inaccurate, boring, and time-consuming. The assessment of young children's national identity is the key breakthrough in improving traditional assessment. It serves an important function in revealing the current situation and characteristics of children's national identity. By being based on the current situation and characteristics, the assessment is effective. Without contextualizing the assessment, focusing only on promoting children's national identity would be an exercise in vain.

Building children's national identity has become the focus of international attention. In this context, it is very necessary to promote children's national identity in China. Scientific and interesting assessment methods should be used to understand the characteristics of children's national identity and promote their national identity. This study investigated whether our specially developed game-based assessment can be employed as a tool to measure children's national identity. Our study provides strong evidence for the application of human-computer interaction in the measurement of children's national identity.

## Literature review

### Young children's national identity

There are a large number of studies and theories on the development of national identity, and the understanding of national identity is becoming advanced (Carrington and Short, 2006; Barrett and Oppenheimer, 2011; Murphy, 2017). The successful creation of national identity is a crucial political process and of core importance for a successful nation-state (Billig, 1995; Liu and Turner, 2018). If individuals are not loyal to their state, the politics (government), military (police and army), and social structures and systems (schools and hospitals) that manage and serve the state will be bereft of their meaning. This flags a core issue on how to determine the status of children's national identity given that children are an important human resource and will determine the future of a state.

Researchers found that national identity gets formed in early childhood, that is, young children can define their own national identities. Nesdale (2004, 2008) proposed the social identity development theory, holding that children can acquire the awareness of belonging to a certain group at the age of three, and begin to pay attention to and prefer the inner group rather than the outer one at the age of four. The research on children's national identity can be traced back to the 1940s and 1950s. Clark and Clark (1940) investigated children's preference for a national image through the study of Black children choosing White dolls but rejecting Black ones. They believed that the skin-color preference is a manifestation of children's national identity. Piaget and Weil (1951) studied Genevan children aged 4–15, believing that children's understanding of the territory and region of the country reflects their comprehension of the country.

Researchers have paid more attention to children's national identity, further clarifying its connotation. Barrett and his colleague systematically expounded on the content framework of children's national identity. They believed that children's national identity is an extremely complex psychological structure, which is composed of two systems: cognition and emotion (Barrett, 2007; Barrett and Oppenheimer, 2011). At the cognitive level, it involves the understanding of the existence of ethnic groups, national geographical territory, national emblem, customs, traditions, historical events, and historical figures symbolically representing the nation, as well as the belief in the typical characteristics of ethnic group members, and the belief in the similarity between self and ethnic types. At the emotional level, the sense of national identity involves many social emotions such as the subjective sense of belonging to the national community, the feelings toward the people who form the national group, and the senses of national pride and national shame, as well as the emotional attachment to the homeland. In addition, Barrett (2000) believes that national identity not only includes cognitive and emotional components but also behavior that forms an important component of national identity. Ashmore et al. (2004) explain that collective identity generally includes self-categorization, evaluation, importance, attachment, a sense of interdependence, social embeddedness, behavioral involvement, content, and meaning. Among them, social embeddedness and behavior participation are emphasized in addition to cognition, emotion, and assessment. Phinney and Ong (2007) held that national identity includes exploration and commitment. The research dimension mainly focuses on the behavior of individuals belonging to a certain nation/country. For example, some individuals spend a lot of time understanding their country. This view has also been recognized by other researchers. To sum up, national identity can include three elements: cognition, emotion, and behavior.

### Game-based assessment

In recent years, technology has been increasingly used for assessments, and among others, intelligent assessment has become an important trend in assessments (Denham et al., 2011; Pelau et al., 2021). Thanks to the development of human-computer interactive technology, game-based assessment (GBA) has emerged in the field of assessment (Kim and Ifenthaler, 2019). Compared with traditional assessments, GBA has many advantages. With the help of human-computer interaction, children can input information through multiple channels such as voice, gesture, eyes, and expression, and the computer can also output multi-channel data such as sounds, images, and videos, which provides a richer, more interesting, and interactive experience for children to participate in the assessment (Singleton et al., 2000; Ellis and Blashki, 2004). With games as the medium, GBA offers more flexibility and higher interest in assessments resulting in a higher involvement of children and reducing their test anxiety and faster data collecting (Wilson et al., 2006b; McCarrick and Li, 2007). With human-computer interaction integrated into daily life, young children are more familiar with and like electronic products and video games (Mumtaz, 2001; Nuutinen et al., 2013; Yousef, 2021). GBA, therefore, has a better environmental application.

Researchers have studied the use of human-computer interactive games to evaluate children's cognitive and non-cognitive abilities. These studies provide methodological enlightenment and reference for the use of human-computer interactive games to evaluate preschool children's national identity. For example, Wilson et al. (2006a) developed a digital contest (The Number Race) to evaluate children's computing ability. Flynn et al. (2019) tested and monitored children in cognitive impairment summer camp, and the completion of single tasks and multi-task was automatically recorded with the help of adaptive algorithm tools. In the non-cognitive field, the Zoo U developed by the 3C Institute (USA) can assess and help children learn interpersonal skills such as impulse control, communication, cooperation, and empathy in the virtual world through man-machine interactive online games (Craig et al., 2016). In addition, Parker et al. (2013) created computerized emotion knowledge measures for preschool children. Game-based assessment has been used to evaluate children's development and has proven to be appropriate for children's assessment. However, there is no game-based assessment to evaluate children's national identity.

### Traditional assessment of children's national identity

Barrett was committed to the research on children's national identity and developed an assessment tool for children, which has been widely used by other researchers. He calculated the strength of the identification scale and investigated the national identity of children aged 5–11 with the help of interviews (Barrett, 2007). This tool has been widely employed by researchers and used to determine the national identity of children in India, Pakistan, Iran, and Germany (Sahlabadi, 2002; Maehr, 2005; Vethanayagam and Barrett, 2007). The interview included six topics and questions such as “which statement do you think best describes you?”, and they were used, respectively, to investigate the degree of the children's identity, pride, importance, feeling, and negative internalization. Barrett et al. (1999) also developed the relative subjective importance task and attribution task. These two task tests belong to traditional forms of assessment where the evaluators carry out tests with the children with the help of relevant materials. Barrett et al. (1999) used the relatively subjective importance task to investigate the attitude of children aged 5–7 toward the importance of national identity. The attribution task posits three situations: positive comparative context conditions (UK and USA), negative comparative context conditions (UK and Germany), and non-comparative conditions (the UK only). This task investigates the judgment of children aged 5–7 on the national image to assess their national identity.

Further, Corenblum (2014), investigated the children's implicit attitudes toward national identity through the implicit association test (IAT) of White-native and native-Black people's tasks. The test included seven test modules in which children could compare the positive and negative characteristics of the concept of state/nation. In addition to the implicit association test, the explicit attitude was also evaluated with the help of a computer. For example, Corenblum (2014) also investigated children's attitudes toward national identity through picture-pointing tasks. The survey questions mainly included the idea of social distance (e.g., “point to the person you want to have lunch with”), group assessment (e.g., “point to the person you have lunch with”), and signs (e.g., “point to someone who looks like a White girl”). The above two assessments (of implicit and explicit attitudes) were computerized judgment tasks that used reaction time as an index, and the participants were required to judge the relationship by the target words.

To sum up, the existing national identity assessments of young children mostly adopt traditional methods. In the process of assessing, children only complete the assessment by choice, and they participate in the assessment in a relatively passive form. The assessment has less interaction and does not evoke children's interest. Computers have been used for assessing children's national identity which has significantly improved assessments in terms of intelligence and convenience, and issues such as measurement errors and subject bias of the traditional assessments can be avoided. However, technology-assisted assessments demand immediacy and rapid reaction. It requires children to have certain cognitive and thinking abilities, and the rapid assessment method is likely to make the process serious and tense, creating a sense of compulsive “examination” experience. Therefore, this assessment method is not available for younger children (3–6 years old).

### The present study

In recent decades, the assessment of human-computer interaction has become one of the most studied topics. As a way of human-computer interaction, game-based assessment is more suitable for assessing young children, making it situational, interesting, and effective. National identity is an important factor affecting the overall development of young children and the identity creation of a country, which has attracted extensive attention from researchers. Nevertheless, assessing young children's national identity continues to be based on traditional evaluation methods, including questionnaires and interviews, which have limitations of being inaccurate, dull, and time-consuming. To understand the characteristics of young children's national identity, it is necessary to use scientific and interesting assessment methods. The present study investigated whether the game-based assessment we developed specifically would be an appropriate tool to measure young children's national identity.

## Materials and methods

### Participants

In this study, preschool children aged 3–6 were included as the research sample. They were drawn from Beijing, Shandong, and Guizhou which feature prominently in the quality of regional preschool education with emphasis on national identity education (e.g., relevant policies) and ample national identity resources (e.g., patriotism education base and national representative cultural landscape). There are differences in the development of preschool children as each provincial administrative region has a different economic level and education quality. To reduce additional differences caused by regions in preschool children's perceptions of national identity, 1–2 cities or regions were selected for research in each provincial administrative region. Based on the principle of easy sampling, cluster sampling was undertaken among kindergarten children. A total of 1,512 preschool children participated in the assessment using human-computer interactive games. Assessments of some children were deemed invalid because they completed the game at <50% or did not spend sufficient game time. So, a total of 1,423 preschool children were included effectively in this study. The specific information relating to preschool children is shown in Table 1. The proportion of children aged 3–3 years and 11 months old, 4–4 years and 11 months old, and 5–5 years and 11 months old were 24.4, 38.8, and 36.8%, respectively. Boys accounted for 58.7%.

**Table 1:** Sample demographics.

**Demographics**	**Frequency**	**Percent**
Region	Beijing	495	34.8%
	Shandong	383	26.9%
	Guizhou	545	38.3%
Child age	3–3 years and 11 months old	347	24.4%
	4–4 years and 11 months old	553	38.8%
	5–5 years and 11 months old	523	36.8%
Child gender	Boys	835	58.7%
	Girls	588	41.3%

A total of 57 preschool children participated in the criterion test. Among them, the proportion of children aged 3–3 years and 11 months old, 4–4 years and 11 months old, and 5–5 years and 11 months old were 30.5, 34.5, and 35.0%, respectively. Girls accounted for 47.8%. In addition, an analysis was done on the reassessment of 62 children's national identity 1 month after the formal assessment. Among the children who participated in the retest, the proportion of children aged 3–3 years and 11 months old, 4–4 years and 11 months old, and 5–5 years and 11 months old were 30.6, 33.9, and 35.6%, respectively. Girls accounted for 45.2% and boys, 54.8%.

### Measures

#### Game-based assessment task

We designed our own GBA task and developed the corresponding game items from the three components of cognition, emotion, and behavior. Based on the characteristics of human-computer interaction, a digital game called Panda Paradise was developed to assess young children's national identity. To the best of our knowledge, this is the first game-based assessment developed to evaluate young children's national identity. The game created a virtual character, the panda, who would lead the young children to complete all the game tasks through verbal and action responses. Children can choose to respond through verbal replies or touch the screen. To add to the fun, the game used cartoon elements, rich colors, and young children's favorite background music. Totally, 35 items were included in the game assessment task. Each item was scored between 0 and 10 points. The higher the score, the higher the level of the child's national identity.

#### Relative subjective importance task

The relative subjective importance task developed by Barrett (2007) was used as the criterion tool. To adapt to the Chinese national context, relevant expressions were modified, for example, “European” was changed to “Asian”. Then, the revised tasks were used in the final test which was delivered by trained researchers in preschool education. Preschoolers were invited to choose the most significant card to describe themselves from a random set of seven cards. Each card held a possible identity for the child and included the child's actual age, gender, state, country, province, race, and religion. To match the literacy level of preschool children, the assessment adopted a combination of text and text and subject language introduction, and let preschool children choose one by one. When the child had chosen a card, it was removed from the pile and the child was asked to choose another card that was second most important to them. This process was repeated until seven cards were selected in seven rounds. The cards were scored 7, 6, 5, 4, 3, 2, 1. Seven was the most important, and 1, was the least important. In the current sample, this task had a Cronbach's alpha of 0.712.

### Procedures

To reduce subject bias, no information including names and nicknames that could identify the identity of preschool children was collected. To avoid measurement error caused by kindergarten details, no information including kindergarten name and class name that could identify the kindergarten were collected in the study. In the testing process, human-computer interactive games were offered on instruments such as educational robots, tablet computers, and smartphones. The test was done in a quiet area of the kindergarten classroom. To ensure the effectiveness of the assessment, kindergarten teachers were clearly informed before the assessment that children were required to complete the game independently. During the assessment, kindergarten teachers could answer questions about equipment operation and rules, but they couldn't guide children in the specific answers to the game assessment. The game time was about 8–12 min.

### Data analysis

After data were collected, SPSS 22.0 statistical software was used for descriptive and statistical tests, correlative analysis, and reliability analysis, and Mplus 8.0 statistical software was employed for confirmatory factor analysis. The Robust Weighted Least Squares estimation for confirmatory factor analysis was more stable and reliable for classified variables. Since the game assessment items of this study were scored between 0 and 10 and belonged to category variables, the Robust Weighted Least Squares estimation was provided in Mplus 8.0 for confirmatory factor analysis. The following indexes recommended by Hu and Bentler (1999) and Kline (2005) were used to evaluate the fitness of the examined model to the data: a comparative fit index (CFI) and Tucker Lewis index (TLI) above 0.95, a standardized root mean square residual (SRMR) below 0.10, and a root mean square error of approximation (RMSEA) below 0.08 were obtained. G^*^power software was employed to conduct sensitivity tests and calculate statistical efficacy.

## Results

### Item analysis

The item analysis was based on the item passing rate, the correlation coefficient between item score and total score, and the critical ratio values. Except for Item 3, other items had a moderate passing rate, and the difficulty of the items fell between 0.349 and 0.698. The results showed that there was a significant positive correlation between all questions and the total score, and the correlation coefficient ranged from 0.424 to 0.698. The participants were grouped into a high-score group (top 27% in the total score) and a low-score group (bottom 27% in the total score). The scores of each item in the high-score group and the low-score group were tested by the *t*-test of independent samples. The critical ratio test showed that except for Item 3, there was a significant difference in the scores of items in both high and low-score groups (*p* < 0.01), indicating that other items were highly distinguishable (see Table 2). Therefore, Item 3 was deleted and the remaining 34 items were retained.

**Table 2:** Results of item analysis.

**Item**	***P***	***R***	**Critical ratio**	**Item**	***P***	***R***	**Critical ratio**
1	0.616	0.613^***^	0.447	19	0.623	0.494^***^	0.665
2	0.412	0.424^***^	0.658	20	0.641	0.523^***^	0.437
3	0.883	0.455^***^	0.313	21	0.622	0.443^***^	0.456
4	0.624	0.586^***^	0.426	22	0.501	0.617^***^	0.443
5	0.694	0.614^***^	0.455	23	0.683	0.503^***^	0.663
6	0.596	0.698^***^	0.441	24	0.628	0.518^***^	0.437
7	0.588	0.693^***^	0.663	25	0.528	0.464^***^	0.446
8	0.349	0.684^***^	0.433	26	0.583	0.636^***^	0.442
9	0.564	0.442^***^	0.453	27	0.591	0.526^***^	0.652
10	0.456	0.561^***^	0.442	28	0.582	0.539^***^	0.426
11	0.528	0.451^***^	0.662	29	0.544	0.467^***^	0.442
12	0.572	0.512^***^	0.439	30	0.567	0.655^***^	0.449
13	0.652	0.671^***^	0.458	31	0.452	0.565^***^	0.658
14	0.686	0.651^***^	0.446	32	0.525	0.551^***^	0.426
15	0.559	0.618^***^	0.666	33	0.593	0.659^***^	0.443
16	0.622	0.615^***^	0.424	34	0.526	0.660^***^	0.446
17	0.634	0.531^***^	0.437	35	0.595	0.443^***^	0.428
18	0.494	0.638^***^	0.447				

*P*, pass rate; *R*, r with the total score,

*** *p* < 0.001 (two-tailed).

### Validity analysis

Random sampling was applied to the samples and half of them were used for exploratory factor analysis and the other half for confirmatory factor analysis. That is, a total of 712 valid data were used for exploratory factor analysis. The adaptability test of the data showed that the value of Kaiser Meyer Olkin (KMO) was 0.871 and Bartlett sphericity test χ^2^ value was 8,195.2, *p* < 0.001, indicating they were suitable for factor analysis. The principal component analysis and the maximal orthogonal rotation of variance were used for factor analysis. Totally three factors with eigenvalues greater than one were extracted from 34 items, the cumulative contribution rate of factors was 61.1%, and the factor load of items was between 0.503 and 0.751. Factor 1 included 15 items in total, Factor 2, 11 items, and Factor 3, 8 items, which was consistent with our theoretical assumptions, indicating the suitability and reliability of the research hypothesis. Combining them with the theoretical assumptions, the three factors were named national cognition mastery, national emotion engagement, and national behavior tendency.

The confirmatory factor analysis was carried out to verify the structural validity of the game assessment. The results show that the model fit well, the item load was more than 0.4, and the fitting indexes were χ^2^/*df* = 329.53, CFI = 0.941, TLI = 0.945, RMSEA = 0.053, and SRMR = 0.042. Except for the chi-square value, which is sensitive due to the large sample size, other fitness indicators were within the acceptable range. In addition, through the correlation analysis between various dimensions, it was found that there was a significant positive correlation among the three dimensions: national cognition mastery, national emotion engagement, and national behavior tendency, with a correlation coefficient of 0.419–0.601 (see Table 3).

**Table 3:** Correlations for three dimensions.

	**1**	**2**	**3**
1. National cognition mastery	–		
2. National emotion engagement	0.601^***^	–	
3. National behavior tendency	0.419^***^	0.518^***^	–

*** *p* < 0.001 (two-tailed).

The relatively subjective importance task was taken as the criterion to test the assessment effect of the game. The results show that the scores obtained by the two assessment methods were significantly positively correlated at the level of 0.01 (bilateral), and the correlation coefficient was 0.453, which shows a medium-level correlation, indicating that the two assessment methods are consistent.

Given the small sample size, this study employed G^*^power software to conduct sensitivity tests and calculate statistical efficacy. The two-tailed test was set in turn, the statistical efficacy was 0.8 and the sample size was 57. The results show that the effect size reached 0.35, which is an acceptable standard. All correlation coefficients can be directly used as the effect quantity. In this study, the correlation coefficient was 0.42, which is significantly higher than the standard of 0.35. Moreover, other settings remained unchanged and the statistical effect increased to 0.95. The results show that the effective amount needs to reach 0.44. The correlation coefficient of this study is close to this standard. To sum up, the results of this study are statistically reliable, that is, the criterion validity test of human-computer interactive games is acceptable.

### Reliability analysis

As shown in Table 4, Cronbach's alpha coefficients in national identity and various fields are higher than 0.8, the split-half reliability is 0.817–0.883, and the test-retest reliability is 0.413–0.645. Among them, given the small retest sample size, the sensitivity test was also carried out through G^*^power software. The results show that all coefficients are within the acceptable range. Therefore, the above research results demonstrated that GBA has higher reliability.

**Table 4:** Results of reliability analysis.

	**Cronbach's alpha coefficients**	**Split-half reliability**	**Test-retest reliability**
National cognition mastery	0.841	0.844	0.645
National emotion engagement	0.925	0.883	0.526
National behavior tendency	0.809	0.817	0.413

## Discussion

The traditional assessment or reaction-time-based (RT-based) computerized task still prevails in the assessment of children's national identity. The traditional assessment is mainly based on others' assessments, and it is highly subjective. It requires evaluators to receive special training, and the collected data has to be coded and scored manually. In addition, it has some limitations, such as insufficient objectivity, time- and labor-consuming, and less participatory. The RT-based computerized assessment has the limitation that it is not suitable for younger children. Compared with the traditional assessment, GBA can make the assessment process more interesting, the assessment results more objective and accurate, and the assessment method more intelligent and efficient (Kim and Ifenthaler, 2019). For these reasons, this study aimed to develop a game to assess preschool children's national identity and verify the suitability of the game-based assessment. Item analysis, reliability test, and validity test were carried out. The results show that the game developed in this study has good item discrimination, high reliability, and validity, so it can be used as a tool to assess children's national identity.

The item analysis shows that the test for the items (exclusive of Item 3) meets the standard. The passing rate of Item 3 is 0.883, that is, 88.3% of preschool children have the correct answer on this item. The item aims to examine preschool children's identification of the Chinese nation, that is, whether they can define they are Chinese in clear and discerning ways. Their passing rate of 88.0% shows that most preschool children can know the country where they live, which reflects the universal awareness of preschoolers about the country's name. However, this item is easier from the perspective of measurement. Items with a high passing rate may have a floor effect. Therefore, this item was deleted from our study while retaining the remaining 34 items.

Exploratory factor analysis showed that after Item 3 was deleted from the original questionnaire, the factor load of the remaining 34 items satisfied the psychometric standards, and the factor attribution was consistent with the hypothetical structure, supporting the three-dimensional division. The results of confirmatory factor analysis show that 34 items have good data fitting in Chinese children, which supports the three-dimension division and proves the high structural validity of the scale from the perspective of metrology. It also showed that the assessment content of human-computer interaction games covered three aspects: national cognition mastery, national emotion engagement, and national behavior tendency.

The assessment effect of human-computer interactive games was tested by the relatively subjective importance task as a criterion. As previously mentioned, Barrett, as a representative researcher who focused on national identity, developed a variety of tools to assess the attribution task and the relatively subjective importance task (Barrett, 2007). Barrett believed that the relatively subjective importance task was a preferred form of assessment for children. Therefore, this study took the relatively subjective importance task as a criterion to test the assessment effect of human-computer interactive games and traditional assessment tools. Given that the study was undertaken during the pandemic-triggered lockdowns, amid suspended classes or the difficulty to enter some kindergartens because of the lockdown restrictions, this study selected three classes of junior, middle, and senior classes in a kindergarten, and a total of 57 preschool children participated in the standard test. The results showed that the scores obtained by the two assessment methods were significantly positively correlated, indicating that the game-based assessment was consistent with the traditional assessment in the research results. That is, the assessment tool we developed can effectively measure children's national identity.

The purpose of this study was to test the effectiveness of the GBA task in assessing children's national identity. The results show that all items of the game we developed have good discrimination ability, and the tests of reliability and validity meet the measurement requirements. GBA can be used as an assessment tool for future research to assess the national identity of Chinese children and can be a powerful supplement to traditional assessment. In short, the revised game has 34 items and applies 2-point scoring. The game has good reliability and validity and can be used as an effective measurement method to evaluate children's national identity.

Against the background of the new era, modern technology injects new vitality into assessments, and games have become a new way of assessing children (Manly et al., 2001; Walczak-Kozłowska et al., 2021). This study developed a game to assess children's national identity, and it serves as a game and an intelligent assessment tool. This tool, with the game as a carrier, actualizes children's participation from passive to active and increases their interest in the assessment. Based on intelligent technology, it can ensure objectivity, efficiency, and convenience of the assessment, and overcome the limitations of traditional assessment, such as lack of objectivity, time and labor intensive, along with time and space constraints. Compared with the existing assessment tools, the human-computer interactive game developed in this study has obvious advantages.

However, this study also has some limitations. First, the subject selection should be improved in representativeness and scale; a representative or larger sample size of subjects is helpful to understand the characteristics of preschool children's national identity more scientifically. The game used in this study can effectively overcome the time and space constraints, and sampling is more convenient due to the limited ability of researchers to undertake the assessment amid the irregular suspension of kindergartens caused by the pandemic. Our study had a small sample size in some tests (such as the criterion test). Although it reached statistical power, a small sample size may lead to a one-sided testing result. Therefore, to effectively and objectively explore the characteristics of preschool children's national identity, future research should design a more rigorous sampling method, and select a range of subjects from urban and rural areas from eastern, central, and western regions. Second, intelligent game design should be strengthened. Although the results of this study show that the currently developed human-computer interactive game presented ideal feedback, there are some challenges due to lack of technical experience in the design of human-computer interactive games, such as insufficient system fluency, low intelligent level, and insufficient game interest. Such virtual reality and augmented reality can be used in future research to make the assessment more interesting and intelligent (Colombo et al., 2019).

## Conclusions

This study aims to examine children's national identity through scientific and interactive evaluation. We specially developed a game-based assessment to test whether this tool is suitable for measuring children's national identity. Item analysis, reliability test, and validity test were carried out. The results show that the game developed in this study has good item discrimination, high reliability, and validity. Overall, the results show that our research provides a reliable assessment tool with 34 items for evaluating children's national identity. The game can be successfully used to assess children's national identity, with acceptable validity and reliability. These findings demonstrate the prospect of game-based assessment in reliably assessing children's national identity.

## Data availability statement

The raw data supporting the conclusions of this article will be made available by the authors, without undue reservation.

## Ethics statement

The studies involving human participants were reviewed and approved by Beijing Normal University. Written informed consent to participate in this study was provided by the participants' legal guardian/next of kin. Written informed consent was obtained from the individual(s), and minor(s)' legal guardian/next of kin, for the publication of any potentially identifiable images or data included in this article.

## Author contributions

All authors listed have made a substantial, direct, and intellectual contribution to the work and approved it for publication.

## Funding

This work was supported by the National Natural Science Foundation of China (Grant No. 62177010) and the International Joint Research Project of Faculty of Education, Beijing Normal University (Grant No. ICER202202).

## Conflict of interest

The authors declare that the research was conducted in the absence of any commercial or financial relationships that could be construed as a potential conflict of interest.

## Publisher's note

All claims expressed in this article are solely those of the authors and do not necessarily represent those of their affiliated organizations, or those of the publisher, the editors and the reviewers. Any product that may be evaluated in this article, or claim that may be made by its manufacturer, is not guaranteed or endorsed by the publisher.
